# Bioactive Compounds in Coffee: Metabolism, Bioavailability and Health Effects—A Review

**DOI:** 10.3390/molecules31091404

**Published:** 2026-04-23

**Authors:** Hajnal Finta, Sándor Pál, Margit Solymár, Zsuzsanna Faust, Marius-Călin Cherecheș, Florina Ruța, Daniela-Edith Ceană, Corneliu-Florin Buicu, Enikő Nemes-Nagy

**Affiliations:** 1 Department of Public Health and Health Management, George Emil Palade University of Medicine, Pharmacy, Science and Technology of Targu Mures, 540142 Targu Mures, Romania; hajnal.finta@umfst.ro (H.F.);; 2 Department of Laboratory Medicine, University of Pécs, H-7633 Pécs, Hungary; margit.solymar@aok.pte.hu (M.S.); faust.zsuzsanna@pte.hu (Z.F.); 3 Department of Drug Industry and Pharmaceutical Management, George Emil Palade University of Medicine, Pharmacy, Science and Technology of Targu Mures, 540142 Targu Mures, Romania; marius.chereches@umfst.ro; 4 Department of Community Nutrition and Food Safety, George Emil Palade University of Medicine, Pharmacy, Science and Technology of Targu Mures, 540142 Targu Mures, Romania; 5 Department of Chemistry and Medical Biochemistry, George Emil Palade University of Medicine, Pharmacy, Science and Technology of Targu Mures, 540142 Targu Mures, Romania; eniko.nemes-nagy@umfst.ro

**Keywords:** chlorogenic acids, coffee antioxidants, bioactive compounds, bioavailability, cardio-metabolic protection, coffee roasting, gut microbiota, melanoidins, metabolism, Nrf2 pathway

## Abstract

Coffee is a very popular psychoactive beverage with a complex composition. Besides its stimulant effect due to caffeine, it contains several bioactive compounds with antioxidant properties and potent metabolic activity. Its clinical efficacy is fundamentally determined by the bioavailability and metabolic fate of its constituents. The bioactive components of coffee, such as polyphenols, melanoidins, phytosterols, biogenic amines, and carotenoids, have notable antioxidant, anti-inflammatory, and immunomodulatory effects. This review aims to present the main bioactive components of coffee, their biological effects, mechanisms of action, and the influence of preparation methods and individual variability on metabolic outcomes in common chronic diseases. The data are synthesized from clinical, prospective, and interventional studies to examine how processing variables and biological metabolism influence the health-promoting potential of coffee antioxidants. Brewing methods like hot filtration optimize the extraction of these antioxidants. Individual clinical outcomes are further modulated by genetic polymorphisms and gut microbiota variability, which influence the activation of the cellular Nrf2 antioxidant defense pathway.

## 1. Introduction

**Figure 1:**
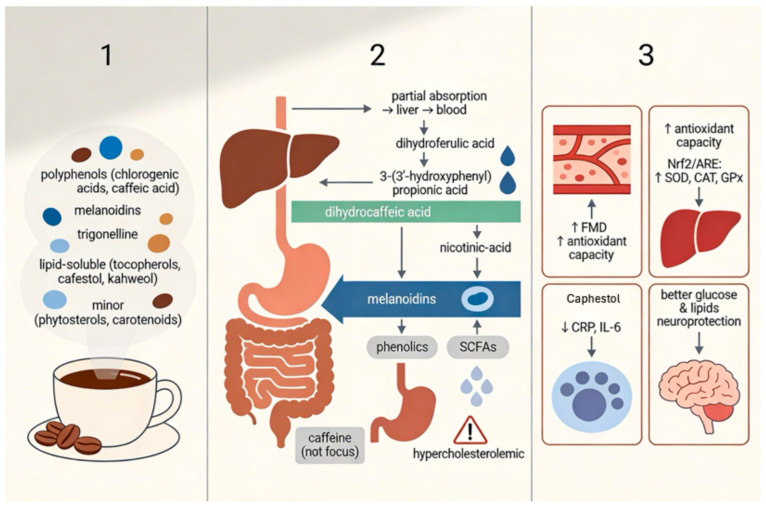
Non-caffeine bioactive substances of coffee, their absorption and biological effects.

Coffee is the most widely consumed beverage globally after water, with significant cultural and dietary relevance in both developed and developing countries [1,2]. Its popularity is driven not only by its stimulant effects, primarily due to caffeine, but also by its complex chemical composition (Figure 1), which includes a wide array of bioactive compounds such as polyphenols, alkaloids, diterpenes, and melanoidins [1,3,4].

Oxidative stress is defined as the imbalance between the production of reactive oxygen species (ROS) and the body’s antioxidant defenses, leading to cellular and molecular damage. This process is implicated in the pathogenesis of chronic diseases, including aging, inflammation, cardiovascular disease, diabetes mellitus, neurodegeneration, and carcinogenesis [5,6].

Dietary antioxidants play a crucial role in counteracting oxidative stress. Coffee is a major source of dietary antioxidants in the Western diet, particularly phenolic compounds such as chlorogenic acids, caffeic acid, and melanoidins, which contribute to its antioxidant capacity [7,8,9,10]. The chemical composition of coffee antioxidants is influenced by bean origin, agricultural practices, and especially by processing methods such as roasting. Roasting alters the levels of chlorogenic acids and generates melanoidins, which have distinct antioxidant and anti-inflammatory properties [9,10,11]. The water used in brewing is not just a passive medium; the minerals dissolved in the water, which differ depending on the source [12], affect how aromatic and soluble compounds are extracted from the beans [13].

In vitro and in vivo studies demonstrate that regular coffee consumption is associated with increased glutathione levels, improved protection against DNA (deoxyribonucleic acid) damage, and reduced biomarkers of oxidative stress and inflammation [11,14,15]. Epidemiological data consistently show that moderate coffee intake (typically 3–5 cups/day) is associated with reduced risk of cardiovascular disease, type 2 diabetes, neurodegenerative disorders, and certain types of cancer [1,2,16,17,18]. Both caffeinated and decaffeinated coffee confer benefits, though the addition of sugar and cream may attenuate these effects [2].

Safety limitations include potential adverse effects with excessive intake, such as anxiety, sleep disturbances, and increased risk in pregnancy (limit caffeine to <200 mg/day) [2,17,19]. Some compounds formed during roasting (e.g., acrylamide) are potentially harmful, but current evidence supports the safety of moderate coffee consumption for most adults [1,3,17,19].

This review aims to provide a comprehensive overview of the main bioactive components of coffee, their biological effects, bioavailability, mechanisms of action, synergistic interactions, and the influence of preparation methods on the profile of active compounds. It also aims to analyze the metabolic effects of coffee consumption in common chronic diseases. Evidence is integrated across multiple levels of investigation, including mechanistic studies, absorption, distribution, metabolism, and excretion (ADME) processes, animal models, human clinical interventions, and epidemiological analyses. Mechanistic and experimental findings elucidate molecular pathways and metabolic transformations, while human and population studies contextualize these mechanisms within the framework of physiological bioavailability and long-term health outcomes, providing a holistic understanding of the metabolic fate and functional effects of antioxidants in coffee.

### Literature Search Strategy

A structured literature search was performed in PubMed, Scopus, and Web of Science using combinations of keywords, including coffee antioxidants, chlorogenic acids, caffeine, trigonelline, melanoidins, oxidative stress, Nrf2 pathway, coffee metabolism, cardiovascular disease, type 2 diabetes, neuroprotection, preparation methods, roast degree, and bioavailability. Studies were included if they investigated coffee composition or antioxidant properties, assessed biological or clinical effects of coffee consumption, examined mechanisms of action or processing effects, and were human clinical, observational, experimental, or systematic reviews published in English-language peer-reviewed journals. Studies focusing on caffeine sources other than coffee, lacking methodological transparency, or lacking translational relevance were excluded. Titles and abstracts were screened for relevance, followed by full-text appraisal. Due to the heterogeneity of study designs, analytical methods, preparation techniques, and outcome measures, the results were synthesized narratively. The evidence was organized by thematic areas, including antioxidant classes, processing effects, bioavailability and metabolism, cellular mechanisms, metabolic and cardiovascular outcomes, neuroprotection, and individual variability. The initial search identified approximately 200 studies published between January 2020 and December 2025, supplemented by prior reference research, with 159 studies included in the final synthesis.

## 2. Major Antioxidants in Coffee

**Figure 2:**
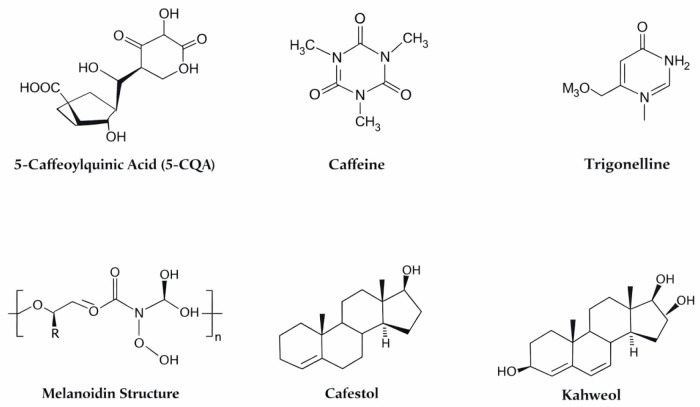
Major Coffee Antioxidants.

Coffee is a complex matrix of bioactive compounds with significant antioxidant properties. The major classes of antioxidants include chlorogenic acids (CGAs), alkaloids (caffeine and trigonelline), melanoidins formed during roasting, and lipid-soluble compounds such as tocopherols and diterpenes [20,21,22,23,24]. The concentration of these compounds differs between the coffee bean and a brewed cup, as extraction efficiency, roast degree, and brewing method strongly influence the final content. These compounds differ in concentration, mechanisms of action, and clinical/translational evidence supporting their biological effects (Table 1, Figure 2) [25].

### 2.1. Chlorogenic Acids (CGAs)

Chlorogenic acids, particularly 5-O-caffeoylquinic acid (5-CQA), are the most abundant phenolic antioxidants in coffee. In addition to 5-CQA, feruloylquinic and dicaffeoylquinic acids have been identified as modulators of oxidative stress, particularly at the mitochondrial level, and as inhibitors of endothelial inflammation. In green coffee beans, CGA content is typically 5–12% of dry weight, whereas brewed coffee contains approximately 70–350 mg/100 mL depending on preparation parameters. The concentrations of CGAs in brewed coffee typically range from approximately 70 to 350 mg/100 mL, with effects that are dose-dependent on markers such as malondialdehyde and protein carbonyls [26]. Recent meta-analyses of randomized, placebo-controlled trials have demonstrated a 15–20% reduction in HOMA-IR (homeostatic model assessment of insulin resistance) among individuals consuming higher CGA doses compared to controls. Mechanistically, CGAs exhibit potent radical scavenging activity, inhibit xanthine oxidase, and activate nuclear factor erythroid 2-related factor 2 (Nrf2)-dependent cellular defense pathways, with 5-CQA showing higher activity compared to other CGAs in both polar and lipid environments (Table 1), [20,21,22]. However, many clinical studies are short term, and optimal dosing for metabolic prevention has yet to reach consensus.

### 2.2. Caffeine and Trigonelline

Caffeine is a major alkaloid in coffee with well-established neuroprotective, anti-inflammatory, and metabolic effects. Recent preclinical evidence (2021–2025) shows that caffeine reduces β-amyloid accumulation and protects microglia in animal models, supporting epidemiologic findings of inverse associations with neurodegenerative disease and type 2 diabetes risk [1,27,28]. Caffeine content ranges from 1 to 2% in green coffee beans and 40 to 120 mg per 100 mL in brewed coffee, depending on the brewing method [29].

Trigonelline, also present in considerable amounts, has been shown to modulate antioxidant gene expression, including Nrf2 and heme oxygenase-1 (HO-1), in vivo, complementing earlier in vitro data. Although human clinical evidence is still limited, traditional dietary exposure suggests a favorable safety profile (Table 1), [27,30]. Limitations include substantial inter-study variability in dosing and preparation methods, and incomplete characterization of bioavailability in humans.

### 2.3. Melanoidins

Melanoidins are high-molecular-weight brown polymers formed during the Maillard reaction in coffee roasting. They contribute significantly to coffee’s overall antioxidant capacity through radical scavenging and α-dicarbonyl trapping and have recently been shown to possess prebiotic activity, stimulating short-chain fatty acid production in the colon. Ex vivo assays have quantified α-dicarbonyl trapping with IC_50_ values between 50 and 120 μg/mL, comparable to or exceeding those of green tea polyphenols. Melanoidins are absent in green coffee beans and are generated during roasting; their concentration in brewed coffee is highly dependent on roast degree and brewing method, with darker roasts yielding higher melanoidin levels [31]. However, most evidence is derived from in vitro or ex vivo studies, and in vivo bioavailability in humans remains poorly defined (Table 1), [3,32,33,34].

### 2.4. Lipid Fraction: Tocopherols, Cafestol, and Kahweol

Coffee’s lipid fraction contains tocopherols (vitamin E) and the diterpenes cafestol and kahweol. Tocopherols contribute modestly to antioxidant capacity but have shown synergistic inhibition of lipoperoxidation when combined with CGAs. Cafestol and kahweol exhibit cellular antioxidant effects in mechanistic studies; however, randomized controlled trials have demonstrated that unfiltered coffee rich in these diterpenes raises LDL cholesterol and liver enzyme levels in a dose-dependent manner, with increases of approximately 10–25% reported. Filtered coffee substantially reduces exposure to these compounds, mitigating hyperlipidemic effects [35,36,37]. Tocopherols and diterpenes are present in both green beans and roasted coffee, but their extractable concentrations in a brewed cup depend strongly on filtration and brewing conditions [38].

**Table 1:** Major antioxidants in coffee.

Compound Class	Typical Concentration in Brewed Coffee mg/100 mL	Mechanisms of Action	Clinical/Translational Evidence	Limitations
**Caffeine** [1,27,30]	~1–2% of green bean mass	Adenosine receptor antagonism; mitochondrial modulation; anti-inflammatory effects	Epidemiologic associations with reduced neurodegenerative and T2D risk	Dose-dependent effects; heterogeneity in studies
**Melanoidins** [3,32,34].	Varies with roast level	Carbonyl trapping; prebiotic modulation (SCFA)	Ex vivo radical and carbonyl trapping	In vivo bioavailability unclear
**Chlorogenic acids (CGAs)**– **5-CQA, 3-,4-diCQA** [20,21,22]	~70–350	Free radical scavenging; metal chelation; xanthine oxidase inhibition; Nrf2 activation	RCTs show improved insulin sensitivity and reduced oxidative markers	Short term; inconsistent dose definitions
**Trigonelline** [27,30].	~0.8–1.05% in green beans	Modulates antioxidant gene expression (Nrf2, HO-1)	In vivo preclinical evidence; limited human data	Few RCTs in humans; variable preparation impact
**Tocopherols (vitamin E)** [35,36,37]	Modest amounts	Radical scavenging; cell membrane protection	Limited specific clinical evidence in coffee context	Contribution small; varies by roast
**Cafestol and Kahweol** [35,37]	Present mainly in unfiltered coffee	Cellular antioxidant actions; modulate phase II enzymes	RCTs show LDL increases; some chemopreventive signals	Adverse lipid effects offset antioxidant benefits

### 2.5. Comparative Contribution of Coffee Antioxidants: Direct vs. Indirect Activity

Coffee’s antioxidant profile reflects a dynamic balance between direct chemical scavenging and indirect cytoprotective mechanisms. CGAs are present at high concentrations in green and lightly roasted beans (~5–12% dry weight) and are partially extracted during brewing (~70–350 mg/100 mL depending on method), while melanoidins are absent in green beans and are generated during roasting, contributing primarily to indirect antioxidant effects in brewed coffee. Caffeine and trigonelline are present in both beans and brewed coffee, with extraction efficiency influencing their final concentrations. CGAs are the predominant contributors to radical scavenging in light-roasted coffee, acting through hydrogen donation and metal chelation. Melanoidins, formed during roasting, function primarily through carbonyl trapping and modulation of gut microbiota, representing indirect antioxidant mechanisms. Caffeine exerts comparatively weak direct radical scavenging but has significant receptor-mediated effects that influence inflammation and mitochondrial function. Importantly, in vivo antioxidant efficacy does not correlate directly with in vitro scavenging assays, as circulating metabolites, tissue distribution, and signaling pathways determine biological outcomes [29,39,40].

### 2.6. Integrated Perspectives

The interactions between coffee’s bioactive constituents may produce synergistic or antagonistic effects that explain heterogeneity in epidemiological findings. The concentrations of these compounds differ between green beans, roasted beans, and brewed coffee, influencing both their direct and indirect biological activities. CGAs and alkaloids are partially extracted during brewing, while melanoidins are generated during roasting and are present primarily in the prepared beverage. These variations determine the exposure of human tissues to bioactive compounds and circulating metabolites, affecting the measured oxidative and inflammatory biomarkers [39,41]. Recent studies employing omics approaches (metabolomics, lipidomics) have linked coffee consumption to specific oxidative and inflammatory biomarkers, advancing our understanding of mechanistic pathways [14] (Table 2).

## 3. Impact of Processing and Brewing

The antioxidant profile of coffee is shaped by the interplay of roasting and brewing, with light-to-medium roasts and hot, pressure-based brewing methods maximizing the concentration and extractable activity of antioxidant compounds in the prepared beverage, while the physiological bioavailability of these compounds depends on absorption and metabolism in the human body. Quantitative differences in antioxidant content exist depending on roast degree and extraction methods (Table 2).

### 3.1. Roasting Degree

Green coffee beans contain the highest levels of chlorogenic acids (CGAs), particularly caffeoylquinic acids, which are heat-labile and undergo significant degradation during roasting. Recent studies confirm that the roasting process does not only cause the degradation of bioactive compounds, but also generates a dynamic balance between their loss and neoformation. Thus, the content of chlorogenic acid, the main antioxidant in raw beans, decreases progressively during roasting, from approximately 34.18 mg/g to 2.58 mg/g after intense roasting at 220 °C, while the concentrations of some derivative compounds, such as gallic acid and caffeic acid, as well as the products of the Maillard reaction (melanoidins with antioxidant activity), increase concomitantly [42]. Melanoidins contribute substantially to the antioxidant activity of roasted coffee, especially at darker roast levels, although their formation does not fully compensate for the loss of CGAs in terms of total antioxidant capacity [43,44,45,46]. Additionally, the structure and molecular weight of melanoidins influence their bioaccessibility and interaction with metal ions, which may modulate oxidative processes in vivo. Recent studies suggest that the balance between CGA degradation and melanoidin formation determines the net antioxidant potential, and this dynamic is also affected by factors such as bean origin, roasting profile, and brewing method. Moreover, CGA-derived metabolites and melanoidins may act synergistically, contributing to both direct radical scavenging and modulation of endogeno us antioxidant pathways [34].

Recent analyses performed using Liquid Chromatography–Mass Spectrometry (LC-MS) techniques show that the degree of roasting significantly influences the phenolic profile, with light roasting being associated with a maximum content of phenolic compounds, medium roasting offering an optimal balance between bioactivity and sensory characteristics, and intense roasting causing a pronounced degradation of phenolic compounds [47]. Light and medium roasts generally preserve more CGAs and exhibit higher antioxidant capacity compared to dark roasts. Multiple assays, including FRAP (Ferric Reducing Antioxidant Power) and ORAC (Oxygen Radical Absorbance Capacity), consistently show that antioxidant activity peaks at light-to-medium roast degrees and declines with further roasting due to progressive CGA degradation. The contribution of melanoidins to antioxidant capacity increases with roast degree, but the overall antioxidant potential is highest when both CGAs and melanoidins are present in significant amounts, as seen in light and medium roasts [9,45,46,47,48,49,50,51,52]. Furthermore, the synergistic interactions between residual CGAs and melanoidins may enhance radical scavenging efficiency, while differences in molecular size and solubility of melanoidins influence their bioaccessibility and functional activity in vivo. Roasting-induced structural changes in CGAs and melanoidins may also modulate their capacity to chelate transition metals and affect redox signaling pathways, contributing to differential antioxidant outcomes depending on roast profile. These results support the current perspective according to which roasting does not simply reduce the antioxidant potential, but remodels the phenolic profile of the final product [42]. Although excessive or uncontrolled frying can favor the formation of compounds with toxicological potential, such as acrylamide, polycyclic aromatic hydrocarbons and advanced glycation end products, common commercial practices generally maintain their levels within limits considered safe, and recent data show that acrylamide reaches maximum concentrations in the early stages of frying and subsequently decreases through thermal degradation, while antioxidant activity is relatively preserved due to the concomitant formation of new bioactive compounds, supporting the current concept according to which the frying process simultaneously generates both potentially harmful molecules and molecules with a protective effect, the final proportion available for physiological activity being further influenced by the preparation method and extraction parameters [49,50,51,52,53]. Recent data indicate a differential sensitivity of bioactive compounds to the roasting process, with caffeine being relatively thermally stable, chlorogenic acids showing increased sensitivity to temperature, while flavonoids undergo changes dependent on the degree of roasting [54].

### 3.2. Brewing Methods

The latest randomized controlled trials and meta-analyses demonstrated that brewing methods significantly influence the clinical outcomes of coffee consumption by altering the levels and bioavailability of key antioxidants. Hot brewing methods (e.g., espresso, hand-filtered, and fully automated machine brews) consistently yield higher concentrations of chlorogenic acids, total phenolics and melanoidins, which are associated with improved endothelial function and reduced cardiovascular risk, as evidenced by significant increases in flow-mediated vasodilation (FMD) in both acute and longer-term studies [55,56,57]. The efficiency of antioxidant extraction is influenced not only by temperature and pressure but also by particle size, brew ratio, and contact time, highlighting the multifactorial nature of bioactive compound availability in vivo. Cold brew methods generally extract less chlorogenic acid and melanoidins but may yield higher caffeine content, depending on steeping time and temperature [58,59,60]. Interestingly, prolonged cold steeping may also lead to selective enrichment of minor phenolic metabolites, potentially modulating neuroprotective and anti-inflammatory effects (Table 2).

Unfiltered brewing methods (e.g., French press, Turkish, Scandinavian boiled coffee) result in higher levels of cafestol and kahweol, which are linked to increased LDL (low density lipoprotein) cholesterol and should be avoided in individuals at risk for hyperlipidemia or cardiovascular disease [46]. Filtered methods (e.g., paper filter, V60) effectively reduce diterpene content, mitigating this risk while preserving antioxidant benefits [56,61,62,63,64,65,66,67,68,69,70] (Table 3). Moreover, filtration may influence the matrix interactions between melanoidins, CGAs, and other phenolics, thereby affecting both absorption kinetics and systemic bioactivity

Neuroprotective effects are primarily attributed to caffeine, chlorogenic acids, and trigonelline, with evidence supporting reduced risk of neurodegenerative diseases and stroke across brewing methods, though the magnitude of effect may be greater with methods that maximize extraction of these compounds [27,56,60]. The highest antioxidant and phenolic content is typically observed in espresso and machine-brewed coffee, while cold brew and percolation-based cold maceration can enhance lipid and trigonelline extraction [56,57,60].

Hot, filtered brewing methods optimize cardiovascular and metabolic benefits by maximizing antioxidant extraction and minimizing diterpene intake, while cold brew and unfiltered methods may increase caffeine and diterpene exposure, respectively, with implications for individual risk profiles [55,56,57,60,61].

## 4. Bioavailability, Metabolism and Cellular Mechanisms

The comparative efficacy and bioavailability of major coffee antioxidants in humans are determined by their chemical structure, absorption kinetics, metabolism, and tissue distribution, which in turn influence clinical outcomes. These effects are further modulated by individual genetic polymorphisms in metabolizing enzymes, diet, and gut microbiota composition, highlighting the inter-individual variability in clinical responses.

### 4.1. Absorption and Metabolism

Chlorogenic acids undergo biphasic absorption and extensive metabolism in the human gastrointestinal (GI) tract. Only a small fraction (approximately 33%) of intact CGAs is absorbed in the small intestine, with the majority reaching the colon where gut microbiota plays a crucial role in their transformation [36,43,51,71,72,73,74]. Recent metabolomics studies have quantified more than 40 distinct colonic CGA metabolites, emphasizing the complex biotransformation network and potential bioactive diversity in systemic circulation [75]. In the stomach and small intestine, CGAs are partially hydrolyzed by esterases to release caffeic, feruloyl, and quinic acids, which are then absorbed and undergo phase II metabolism (sulfation, glucuronidation, methylation) to form circulating conjugates [60,71,72]. These metabolites exhibit antioxidant and anti-inflammatory effects, with clinical trials showing acute improvements in plasma antioxidant capacity and endothelial function after CGA-rich coffee consumption [43,72,76,77,78,79].

Colonic metabolism by gut microbiota is the predominant pathway for CGA biotransformation. The gut microbiota rapidly degrades CGAs (within 1–6 h) through a series of enzymatic reactions, including ester hydrolysis, reduction in the aliphatic double bond, dehydroxylation, and ring fission [80,81,82]. The major bioavailable metabolites produced by colonic bacteria include dihydrocaffeic acid, dihydroferulic acid, and 3-(3′-hydroxyphenyl) propionic acid, which comprise 75–83% of total catabolites and are efficiently absorbed into systemic circulation [81,82,83]. These colonic metabolites exhibit delayed kinetics (peak at 8–12 h post-consumption) and represent the most abundant group of coffee phenolic metabolites in plasma and urine [84,85]. These delayed metabolites may exert prolonged biological activity, including modulation of endothelial function and gut-liver axis signaling, which could contribute to long-term health benefits.

Caffeine is rapidly and almost completely absorbed, with peak plasma levels within 15–120 min and a half-life of 2.5–4.5 h in adults. It distributes widely, crosses the blood–brain barrier, and is metabolized by hepatic CYP1A2 to paraxanthine, theobromine, and theophylline, which also possess antioxidant properties [1,86]. Trigonelline is absorbed early and persists in plasma with a half-life of ~5 h. It is metabolized to nicotinic acid and other derivatives, contributing to antioxidant and neuroprotective effects [84,85,86,87]. Melanoidins are poorly absorbed in the upper GI tract but reach the colon, where they are fermented by gut microbiota, releasing phenolic compounds and short-chain fatty acids (SCFAs) [88,89,90,91,92]. SCFAs produced during fermentation also act as signaling molecules that regulate inflammation and gut barrier integrity, linking coffee intake to systemic metabolic effects [93,94]. Recent studies indicate that phenolic compounds in roasted coffee remain bioaccessible after digestion and can modulate the gut microbiota by stimulating SCFA production, and in vitro and in vivo analyses show that light to medium roasting degrees are associated with increased hepatic antioxidant enzyme activity and reduced expression of proinflammatory markers, while intense roasting diminishes these beneficial biological effects [47,51].

### 4.2. Cellular Mechanisms—The Nrf2 Pathway

Beyond direct radical scavenging, coffee components activate the pathway, the master regulator of cellular antioxidant responses. Activation of Nrf2 by coffee components also upregulates cytoprotective genes involved in phase III transport and mitochondrial function, which may synergize with direct antioxidant effects [95]. Chlorogenic acids, N-methylpyridinium (a roasting product), and other coffee phenolics induce Nrf2 nuclear translocation and activate antioxidant response element (ARE)-dependent gene expression, leading to upregulation of endogenous antioxidant and phase II detoxification enzymes [7,93,94].

Animal and human studies demonstrate that coffee consumption significantly increases the activity of key antioxidant enzymes: superoxide dismutase (SOD) by 74.8%, catalase (CAT) by 59.4%, and glutathione peroxidase (GPx) by 135.2%, along with increased Nrf2 levels (131.3%) and total antioxidant capacity (25.1%) [96,97]. These effects occur both acutely and chronically, with dose-dependent responses and long-lasting protection against oxidative stress [15,98,99]. Furthermore, modulation of Nrf2 signaling by coffee has been associated with anti-inflammatory gene expression and improved mitochondrial biogenesis in preclinical models, indicating a broader cytoprotective role beyond classical antioxidant defense [7].

## 5. Individual Variability and Synergistic Interactions

### 5.1. Genetics and Microbiota Variability

Individual variability in gut microbiota composition and genetic polymorphisms, such as Nrf2 and phase II enzyme variants, substantially influence the bioavailability and clinical efficacy of coffee antioxidants [100]. The gut microbiota is the primary driver of inter- and intra-individual differences in the metabolism of coffee-derived phenolics [101]. Genetic polymorphisms in the Nrf2 (NFE2L2) gene, particularly promoter SNPs (Single Nucletide Polymorphisms) such as -617C/A, are associated with reduced basal and inducible Nrf2 transcriptional activity. However, coffee consumption can still induce Nrf2 gene transcription even in SNP carriers, though the magnitude of response is genotype-dependent [102]. Similarly, null genotypes in phase II enzymes (e.g., GSTT1) modulate the induction of detoxification pathways by coffee [93]. Moreover, epistatic interactions between Nrf2 variants and phase II enzyme polymorphisms may further influence individual responsiveness to coffee-derived antioxidants, highlighting the complex gene–nutrient interplay [103].

Targeted modulation of gut microbiota through probiotics (e.g., *Lactobacillus plantarum*, *Bifidobacterium longum*) and prebiotics (e.g., inulin-type fructans) can enhance the bioavailability and efficacy of coffee antioxidants by improving the production of bioactive colonic metabolites [49,73,74,80,81,82,83,87], given the presence of dysbiosis in a large number of individuals [104,105,106,107]. This microbial enhancement is particularly relevant for individuals with Nrf2 polymorphisms, as increased metabolite production can compensate for reduced genetic capacity to activate antioxidant pathways [103]. Additionally, synergistic interactions between coffee metabolites and probiotic-derived metabolites may potentiate Nrf2 activation and antioxidant enzyme expression, providing a mechanistic basis for personalized dietary interventionsm [108].

### 5.2. Dietary Synergy

Coffee consumption interacts synergistically with other dietary polyphenols, such as those from tea, cocoa, and berries, to enhance antioxidant capacity via shared metabolic pathways in the gut microbiota [7,109,110]. Emerging evidence suggests that co-consumption may also influence the bioavailability and systemic distribution of polyphenolic metabolites, leading to enhanced plasma antioxidant levels and improved modulation of redox-sensitive signaling pathways [111].

Co-consumption leads to increased production of SCFAs and phenolic metabolites, modulating gut microbiota composition and function to support anti-inflammatory profiles [106,107]. For example, cocoa and coffee polyphenols undergo cometabolism, producing metabolites that exert additive or synergistic effects on host physiology [107,108]. These metabolites can interact with cellular targets, including Nrf2, NF-κB, and sirtuin pathways, amplifying cytoprotective and anti-inflammatory responses. The synergistic enhancement of health outcomes is mediated by convergent activation of cellular defense mechanisms [7,106,107,108].

## 6. Coffee and Metabolic Health—Type 2 Diabetes and Obesity

Habitual coffee consumption is consistently associated with a reduced risk of type 2 diabetes mellitus (T2DM) [112]. Meta-analyses of over 1.1 million participants found a 6% reduction in T2DM risk for each additional cup of coffee per day, with the highest consumption categories showing a pooled relative risk of 0.71 [113]. Both caffeinated and decaffeinated coffee demonstrate similar protective effects [114]. These associations remain significant after adjustment for major confounders such as body mass index, physical activity, and dietary patterns, suggesting that coffee-derived bioactive compounds may exert independent metabolic benefits. Prospective cohort studies also indicate dose–response relationships across diverse populations, supporting a consistent epidemiological link between coffee intake and improved glycemic outcomes [115].

### 6.1. Mechanisms Related to Glucose Metabolism

Chlorogenic acids (CGAs) improve glucose metabolism by inhibiting hepatic glucose-6-phosphatase, competitive inhibition of intestinal glucose absorption, and enhancement of GLP-1 (glucagon-like peptide-1) secretion [112]. Additionally, CGAs may modulate glucose homeostasis through activation of AMP-activated protein kinase (AMPK), which enhances insulin signaling and promotes glucose uptake in skeletal muscle and adipose tissue [116,117]. Coffee extracts also demonstrate potent α-glucosidase inhibitory activity, which delays carbohydrate digestion and reduces postprandial glucose spikes [118,119,120]. Long-term exposure to coffee bioactives may also protect pancreatic β-cells by reducing oxidative damage and supporting mitochondrial function, thereby contributing to sustained insulin secretory capacity [121,122]. While caffeine acutely reduces insulin sensitivity, habitual consumption leads to tolerance and overall improvements in insulin sensitivity and beta-cell function [123,124].

### 6.2. Anti-Inflammatory Effects on Adipose Tissue

Coffee exerts significant anti-inflammatory effects on adipose tissue, which partially mediate its protective effects against T2DM. Higher consumption is associated with lower concentrations of CRP (C-reactive protein) (−16.6%), IL-6 (interleukine-6) (−8.1%), and higher adiponectin levels (+9.3%). These biomarker changes reflect a shift toward a more favorable adipokine profile that improves insulin sensitivity and systemic metabolic regulation [124,125,126]. Coffee phenolics counteract TNF-α (tumor necrosis factor-α)-induced inflammation in adipocytes by reducing pro-inflammatory cytokines while increasing glutathione expression [127]. Animal studies show that coffee prevents high-fat diet-induced obesity, reduces macrophage infiltration in adipose tissue, and preserves brown adipose tissue thermogenic capacity [128]. Preservation of brown adipose tissue activity may contribute to improved metabolic flexibility and enhanced energy expenditure [129]. Additional mechanisms include regulation of transcription factors involved in adipocyte differentiation, such as PPARγ and C/EBPα, which play central roles in adipose tissue development and lipid storage [130]. Coffee also inhibits adipogenesis by interrupting insulin signaling through the downregulation of IRS1 (insulin-receptor substrate 1) [131].

### 6.3. Obesity and Weight Management

The relationship of coffee consumption with obesity is modest; meta-analyses show small inverse associations with BMI (body mass index) and waist circumference [132]. These associations are thought to result from a combination of metabolic, behavioral, and neuroendocrine effects related to caffeine and other coffee bioactives. Increasing unsweetened caffeinated coffee intake is associated with reduced 4-year weight gain, though adding sugar counteracts this benefit [133]. Moderate consumption is linked to favorable changes in total and visceral body fat [134,135]. Visceral adiposity is particularly relevant because of its strong association with cardiometabolic risk and insulin resistance. Caffeine may improve energy balance by increasing basal metabolic rate and thermogenesis, though clinical weight management impacts remain modest. Mechanistically, caffeine stimulates sympathetic nervous system activity and increases catecholamine release, promoting lipolysis and fatty acid oxidation [135].

## 7. Cardiovascular Protection and Endothelial Function

Moderate filtered coffee intake improves endothelial function via hydroxycinnamic acid-mediated NO (nitric oxide) production, protects against LDL oxidation, and lowers systemic inflammation. These cardioprotective effects are attributed to the combined action of coffee polyphenols, caffeine, and other bioactive compounds that influence oxidative stress, vascular tone, and inflammatory signaling pathways. Emerging evidence suggests that these compounds interact with redox-sensitive transcription factors and endothelial signaling cascades, thereby contributing to improved vascular homeostasis [135].

### 7.1. Vascular Health

Hydroxycinnamic acids found in coffee enhance endothelial nitric oxide synthase (eNOS) activity, leading to increased NO bioavailability. Randomized controlled trials show that chlorogenic acid-rich coffee significantly increases FMD by 1.5–2.5 percentage points over placebo. This improvement in endothelial-dependent vasodilation reflects enhanced vascular responsiveness and reduced oxidative inactivation of nitric oxide. Chlorogenic acids may also improve endothelial function by attenuating NADPH oxidase–derived reactive oxygen species and by modulating intracellular signaling pathways involved in vascular relaxation. Additionally, coffee polyphenols may protect endothelial cells from oxidative damage and improve mitochondrial function, further supporting vascular integrity and microcirculatory regulation [136,137,138].

### 7.2. Lipid Profile and Inflammation

Coffee’s impact on lipid profile is preparation-dependent. Unfiltered coffee raises LDL cholesterol due to cafestol, a potent agonist of the farnesoid X receptor. This difference is largely explained by the retention of diterpenes in paper filters, which significantly reduces circulating levels of cafestol and kahweol after consumption [139]. In contrast, filtered coffee does not associate with adverse lipid changes [139]. Coffee polyphenols provide anti-atherogenic effects by inhibiting LDL oxidation. Inhibition of LDL oxidation is a key mechanism in preventing the formation of atherogenic oxidized LDL particles that promote endothelial dysfunction and foam cell formation within arterial walls [140,141]. Habitual intake is linked to lower plasma concentrations of CRP and IL-6, independent of caffeine content. These reductions in inflammatory biomarkers suggest that coffee consumption may attenuate chronic low-grade inflammation, a central driver of atherosclerosis and cardiometabolic disease [141].

### 7.3. Cardiovascular Effect

Comprehensive reviews, including an umbrella review of over 200 meta-analyses, confirmed that the most significant relative risk reductions were observed with a consumption of 3–4 cups of coffee per day, CVD (cardiovascular disease) mortality showed a 19% decrease [142]. These findings support a nonlinear dose–response relationship in which moderate consumption provides maximal cardiovascular benefit, while extremely high intake does not confer additional protection. Potential mechanisms underlying these epidemiological observations include improved endothelial function, reduced oxidative stress, enhanced insulin sensitivity, and modulation of inflammatory pathways. Collectively, these mechanisms contribute to the observed associations between moderate coffee consumption and reduced risk of coronary heart disease, stroke, and cardiovascular mortality [143].

## 8. Neuroprotection—Coffee and the Aging Brain

Epidemiological evidence shows a strong inverse association between coffee consumption and the risk of neurodegenerative diseases. These associations are supported by converging experimental, clinical, and mechanistic data indicating that coffee bioactive compounds influence multiple pathways involved in neuronal survival, oxidative stress regulation, and neuroinflammation.

Caffeinated coffee intake is consistently associated with a lower risk of Parkinson’s disease, with hazard ratios ranging from 0.18 to 0.85 in men [144]. Sex-specific differences observed in epidemiological studies may be partially explained by hormonal modulation of caffeine metabolism and adenosine receptor signaling, which influence dopaminergic neurotransmission [145].

Adenosine A2A receptor antagonism by caffeine is central to neuroprotection in Parkinson’s, reducing neuroinflammation and dopaminergic neuron loss. Blockade of A2A receptors also modulates glutamatergic neurotransmission and reduces excitotoxicity, a key contributor to neuronal degeneration. Furthermore, A2A receptor inhibition enhances mitochondrial efficiency and reduces oxidative stress within dopaminergic neurons [146,147]. For Alzheimer’s, mechanisms include protection against beta-amyloid toxicity and inhibition of tau hyperphosphorylation, mediated by caffeine and polyphenols [144]. Coffee polyphenols may additionally interfere with amyloid aggregation processes and promote clearance pathways through activation of proteostatic mechanisms [148].

Recent studies have highlighted emerging new mechanisms, including caffeine modulation of α-Syn degradation with enhanced autophagy and caffeine modulation of gut microbiota and the gut–brain axis in Parkinson`s disease [149]. Alterations in gut microbiota composition may influence neuroinflammation through microbial metabolite signaling, including short-chain fatty acids that regulate microglial activation and blood–brain barrier integrity [149].

Bioactive compounds like pyrocatechol suppress neuroinflammation by inhibiting NF-κB (nuclear factor-kappa B) signaling in microglia. Inhibition of NF-κB signaling reduces the production of pro-inflammatory cytokines and reactive oxygen species, thereby limiting chronic neuroinflammatory responses implicated in neurodegenerative progression [150,151,152,153,154].

Mendelian randomization (MR) studies provide mixed results regarding causality. Genetically predicted higher plasma caffeine levels indicate lower risk of Alzheimer’s and Parkinson’s, though some MR studies report no causal association or even a possible increased risk, highlighting the complexity of genetic instruments. These discrepancies may reflect pleiotropic genetic effects, variability in caffeine metabolism genes such as CYP1A2, and differences between lifelong genetic exposure and behavioral consumption patterns [155].

The effect is primarily attributed to caffeine, as decaffeinated coffee does not confer similar protection [1,138,139]. For Alzheimer’s disease, higher intake of unsweetened, caffeinated coffee (≥3 cups/day) is associated with a reduced risk (HR—hazard ratio: 0.75), while no benefit is seen for sweetened coffee [156,157,158]. This observation suggests that metabolic factors such as glycemic load and insulin signaling may modify neuroprotective outcomes, reinforcing the importance of overall dietary context in cognitive aging [159,160,161,162,163].

## 9. Risk of Genetic Variability CYP1A2 and Risk of High Caffeine Intake

### 9.1. Caffeine Sensitivity, Gastrointestinal Issues and Ergogenic Effects

Coffee stimulates gastric acid secretion and may exacerbate symptoms in patients with GERD (gastroesophageal reflux disease) or gastritis. This effect is mediated not only by caffeine but also by other coffee constituents that stimulate gastrin release and gastric secretory activity, potentially influencing lower esophageal sphincter tone in susceptible individuals [140,159,162,163].

Genetic variability in CYP1A2 and ADORA2A affects caffeine sensitivity; slow metabolizers are at increased risk for insomnia, anxiety, and palpitations. Polymorphisms in CYP1A2 influence hepatic caffeine clearance rates, resulting in prolonged plasma caffeine exposure and enhanced stimulation of central nervous system and cardiovascular responses in slow metabolizers. Variants in ADORA2A further modulate individual susceptibility through altered adenosine receptor signaling, affecting sleep regulation, anxiety responses, and perceived stimulant effects [164,165,166,167,168,169,170]. Although genetic testing has limited routine clinical utility, individualized assessment is recommended [155].

According to an investigation, while CYP1A2 genotypes may impact the ergogenic effects of caffeine to some degree, there may be other factors influencing physical performance and physiological response [164,165,166].

Caffeine has been shown to enhance neuromuscular performance by improving skeletal muscle contractile properties, including reduced contraction time and increased maximal muscle displacement, suggesting both peripheral and central contributions to its ergogenic effects [171]. These physiological responses may improve muscle responsiveness and support enhanced physical performance during high-intensity or repeated muscular efforts.

Furthermore, caffeine exerts ergogenic effects along the neuromuscular pathway, from central nervous system stimulation to motor unit recruitment and excitation–contraction coupling within skeletal muscle fibers. Through antagonism of adenosine receptors and increased neurotransmitter release, caffeine may enhance voluntary force production, reduce perceived fatigue, and improve neuromuscular efficiency, supporting its role as an ergogenic aid in both trained and recreational individuals [172].

### 9.2. The Risk of High Caffeine Intake in the Pregnancy and Contaminants

Consensus guidelines recommend restricting caffeine intake to <200 mg/day during pregnancy and lactation to avoid risks of miscarriage and low birth weight [173].

Regarding toxicological contaminants, acrylamide is formed during roasting, and mycotoxins may contaminate poorly stored beans; mitigation includes optimizing roasting and storage conditions [174,175]. Acrylamide formation depends on roasting temperature and duration through Maillard reaction pathways, while proper post-harvest drying, humidity control, and quality monitoring reduce the risk of ochratoxin A contamination. Current exposure assessments indicate that levels in commercially regulated coffee generally remain within established safety thresholds when good manufacturing practices are followed [176,177,178].

## 10. Valorization of Coffee Waste—Spent Coffee Grounds

Spent coffee grounds (SCG) retain substantial amounts of bioactive compounds, including chlorogenic acids and caffeine. In addition to these compounds, SCG contain dietary fiber, proteins, lipids, and phenolic polymers such as melanoidins, which contribute to their functional and nutraceutical potential. The residual antioxidant capacity of SCG depends on roasting degree, extraction efficiency during brewing, and storage conditions prior to processing [179,180,181,182,183]. Extraction methods like subcritical water or deep eutectic solvents can efficiently recover these compounds for use in dietary supplements. These green extraction technologies improve extraction yield while reducing solvent toxicity and environmental impact, supporting sustainable recovery of high-value bioactives. Optimization of extraction parameters, including temperature, pressure, and solvent polarity, further enhances phenolic recovery and biological activity [184]. SCG extracts demonstrate robust free radical scavenging capacity and prebiotic activity, supporting beneficial gut microbiota. Fermentable polysaccharides and melanoidins present in SCG may selectively stimulate the growth of beneficial bacterial genera, contributing to short-chain fatty acid production and improved gut metabolic function. Additionally, SCG-derived compounds have shown antimicrobial and anti-inflammatory properties in experimental models, suggesting broader applications in functional foods and nutraceutical formulations [59,184,185,186]. Valorization of SCG aligns with circular bioeconomy principles, though safety assessments for contaminants remain necessary. Potential safety concerns include accumulation of heavy metals, pesticide residues, and processing-derived compounds, emphasizing the importance of standardized quality control and toxicological evaluation prior to human consumption [182,183].

## 11. Coffee Consumption and Associated Rituals

Beyond its role as a primary source of caffeine, coffee has important social significance, being associated with daily rituals (e.g., drinking in the morning to stimulate alertness or in social contexts to facilitate interaction) [1]. These consumption rituals represent learned behavioral patterns that may influence psychological well-being, circadian regulation, and cognitive performance through conditioned associations between sensory cues and neurophysiological responses. Regular coffee rituals are also linked to social bonding and structured daily routines, factors known to support mental health and perceived quality of life [187].

These rituals vary significantly between cultures, but often include moments of relaxation, socialization, or a break at work, and may contribute to psychosocial well-being. From a behavioral health perspective, coffee consumption rituals may influence stress regulation through social interaction, conditioned relaxation responses, and modulation of neuroendocrine pathways associated with mood and cognitive performance [188].

Consumption rituals, such as adding sugar or cream, can attenuate the beneficial effects of coffee. Associating coffee consumption with smoking, for example, is detrimental to health, and it is recommended to avoid smoking both in general and in combination with coffee consumption. Co-consumption behaviors may modify metabolic outcomes by increasing caloric intake, glycemic load, or exposure to harmful compounds, thereby confounding epidemiological associations between coffee intake and health outcomes. Consideration of lifestyle context is therefore essential when interpreting the health effects of coffee consumption [2,189].

In addition, coffee is often used to increase productivity and alertness, being an integral part of the daily routine for many adults [1,2]. The alertness-promoting effects are primarily mediated by caffeine-induced antagonism of adenosine receptors, which enhances dopaminergic and noradrenergic signaling involved in attention, motivation, and executive function. Furthermore, habitual consumption within predictable daily contexts may reinforce behavioral reinforcement mechanisms and improve task engagement, although excessive reliance on caffeine for performance may contribute to sleep disruption in sensitive individuals [190]. However, the magnitude of these physiological and cognitive responses to caffeine varies considerably between individuals due to differences in caffeine metabolism and genetic polymorphisms affecting enzymes and receptors involved in caffeine pharmacokinetics and pharmacodynamics [191,192,193,194].

### Future Perspectives: Machine Learning in Coffee Bioactivity Research

The integration of artificial intelligence methods into biomedical research has enabled a more precise evaluation of the relationships between bioactive compounds and clinical parameters. The use of machine learning algorithms in the analysis of preclinical data and health questionnaires facilitates the identification of metabolic and behavioral patterns that would otherwise remain undetected through traditional statistical approaches. Such methodologies can contribute to characterizing how individual variability influences the response to coffee metabolites and can support the development of predictive models regarding their biological effects. In this context, the study conducted by Avram et al. demonstrates the utility of algorithm-assisted assessment in evaluating health profiles, providing a methodological framework that can also be applied to investigating the impact of coffee bioactive compounds on metabolic risk [195,196].

## 12. Limitations

Key limitations of this review include study heterogeneity, residual confounding in observational analyses, variations in coffee types and brewing methods, and potential influences of co-consumption factors (e.g., sugar or cream). Future research should aim to clarify where evidence is robust versus uncertain or context-dependent, which would help improve the overall scientific balance.

## 13. Conclusions

Moderate coffee consumption (3–4 cups/day) is generally beneficial for oxidative stress reduction and the prevention of obesity, chronic metabolic and neurodegenerative diseases in healthy adults and it is also linked to reduced all-cause mortality. Important knowledge gaps remain, particularly the need for more long-term human intervention trials (in vivo) to complement observational data and clarify the causal mechanisms of specific bioactive metabolites.

Current literature highlights both the positive impact of moderate coffee consumption on health and its central role in daily habits and rituals, contributing to psychosocial well-being.

Overall, the evidence discussed in this review integrates mechanistic insights, ADME-related processes, experimental models, and human studies, collectively supporting a comprehensive understanding of the bioavailability and metabolic fate of coffee antioxidants.

## Figures and Tables

**Table 2:** Approximate antioxidant retention across coffee roast degrees and brewing methods.

Roast/Brew	% CGA Retained	Total Antioxidant Capacity (FRAP/ORAC)	Notes
Green	100%	100%	Maximizes CGA; no melanoidins
Light roast	70–90%	90–95%	CGA + initial melanoidins
Medium roast	50–70%	85–90%	CGA + increasing melanoidins
Dark roast	<40%	70–80%	CGA low; melanoidins high
Espresso	60–80%	90–95%	High antioxidant and phenolic extraction
Filtered (hot)	50–70%	85–90%	Reduces diterpenes; preserves antioxidants
Cold brew	30–50%	70–80%	Lower CGA; higher caffeine; lipid extraction depends on steeping

**Table 3:** Impact of brewing methods on coffee bioactive compounds and LDL-associated effects [58,59,60,61,62,63,64,65,66].

Brewing Method	Extraction Temp (°C)	Extraction Time	CGAs (mg/100 mL)	Caffeine (mg/100 mL)	Trigonelline (mg/100 mL)	Melanoidins (mg/100 mL)	Tocopherols (mg/100 mL)	Cafestol/Kahweol (mg/100 mL)	Clinical Impact (LDL/Other)
Espresso	90–95	20–30 s	60–80	90–120	30–50	50–100	0.1–0.3	0.5–1.0/0.3–0.6	High antioxidant; moderate diterpene
Filtered (Paper)	90–96	3–5 min	50–70	80–100	25–45	40–80	0.1–0.2	0.1–0.3/0.05–0.2	Preserves antioxidants; low LDL risk
French Press (Unfiltered)	90–96	4–5 min	50–70	80–110	25–45	40–90	0.1–0.3	1.0–2.5/0.5–1.5	Higher LDL association
Cold Brew (Steep)	20–25	12–24 h	30–50	100–140	20–35	20–50	0.05–0.1	0.05–0.1/0.02–0.05	Lower antioxidant; variable diterpene

## Data Availability

The data supporting the findings of this study are available from the corresponding author upon reasonable request.

## Abbreviations

The following abbreviations are used in this manuscript:

ADME	Absorption, distribution, metabolism, and excretion
AGEs	Advanced Glycation End-products
ARE	Antioxidant response element
CAT	Catalase
CGAs	Chlorogenic acids
5-CQA	5-O-caffeoylquinic acid
CRP	C-reactive protein
CVD	Cardiovascular disease
DNA	Deoxyribonucleic acid
eNOS	endothelial nitrix oxide synthase
FMD	Flow-mediated vasodilation
FRAP	Ferric Reducing Antioxidant Power
GERD	Gastroesophageal reflux disease
GI	Gastrointestinal
GLP-1	Glucagon-like peptide-1
GPx	Glutathione peroxidase
HOMA-IR	Homeostatic Model Assessment of Insulin Resistance
HR	Hazard ratio
IL-6	Interleukine-6
IRS1	Insulin receptor substrate 1
LDL	Low density lipoprotein
MR	Mendelian randomization
NF-kB	Nuclear factor-kappa B
NO	Nitric oxide
NrF2	Nuclear factor erythroid 2-related factor 2
ORAC	Oxygen Radical Absorbance Capacity
ROS	Reactive Oxygen Species
SCFAs	Short-chain fatty acids
SCG	Spent coffee grounds
SNPs	Single nucleotide polymorphisms
SOD	Superoxide dismutase
T2DM	Type 2 diabetes mellitus
TNF-α	Tumor necrosis factor-α
